# The Cedar Project WelTel mHealth intervention for HIV prevention in young Indigenous people who use illicit drugs: study protocol for a randomized controlled trial

**DOI:** 10.1186/s13063-016-1250-3

**Published:** 2016-03-09

**Authors:** Kate Jongbloed, Anton J. Friedman, Margo E. Pearce, Mia L. Van Der Kop, Vicky Thomas, Lou Demerais, Sherri Pooyak, Martin T. Schechter, Richard T. Lester, Patricia M. Spittal, The Cedar Project Partnership

**Affiliations:** School of Population and Public Health, Faculty of Medicine, University of British Columbia, 2206 East Mall, Vancouver, BC V6T 1Z3 Canada; The Cedar Project, Centre for Health Evaluation and Outcome Sciences, St. Paul’s Hospital, 588-1081 Burrard Street, Vancouver, BC V6Z 1Y6 Canada; Department of Public Health Sciences, Karolinska Institutet, Tomtebodavägen 18a, Campus Solna, Stockholm, 171 77 Sweden; Division of Infectious Diseases, Faculty of Medicine, University of British Columbia, 566-828 West 10th Avenue, Vancouver, BC V5Z 1L8 Canada; Vancouver Native Health Society, 455 Hastings Street E, Vancouver, BC V6A 1P5 Canada; Canadian Aboriginal AIDS Network, 6520 Salish Drive, Vancouver, BC V6N 2C7 Canada; Neglected Global Diseases Initiative, Faculty of Medicine, University of British Columbia, 564-828 West 10th Avenue, Vancouver, BC V5Z 1L8 Canada

**Keywords:** mHealth, Indigenous, HIV prevention, Substance use

## Abstract

**Background:**

Despite successes in preventing and treating HIV, Indigenous people in Canada continue to face disproportionately high rates of HIV infection. Programs that support healing from lifetime trauma, support connection to culture, and reduce drug-related harms are critical to preventing HIV among young Indigenous people who use drugs. The Cedar Project WelTel mHealth intervention proposed here is a structured mobile-phone initiative to connect young Indigenous people who use drugs with Cedar Case Managers in a community-based setting. The intervention consists of a package of supports, including a mobile phone and cellular plan, weekly two-way text messaging, and support from Cedar Case Managers.

**Methods:**

The Cedar Project WelTel mHealth study is a multi-site Zelen pre-randomized trial to measure the effect of a two-way supportive text-message intervention to reduce HIV vulnerability among young Indigenous people who use illicit drugs in two Canadian cities. The trial is nested within the Cedar Project, an ongoing cohort study addressing HIV and hepatitis C vulnerability among young Indigenous people who use drugs in Vancouver and Prince George, British Columbia. The Cedar Project Partnership, an independent body of Indigenous Elders, leaders, and health/social service experts, governs all aspects of the study. Two hundred participants will be followed over a 16-month period, with HIV propensity score at 6 months as the primary outcome. Secondary outcomes include HIV propensity at 1 year, HIV risk, resilience, psychological distress, access to drug-related services, and connection to culture measured at 6 months and 1 year. Primary analysis is by intention to treat.

**Discussion:**

Culturally safe interventions that address barriers to HIV prevention while supporting the strength of young Indigenous people who use drugs are urgently needed. Despite presenting a tremendous opportunity to connect young, highly transient Indigenous people who use drugs to prevention services, supportive two-way mHealth programs have yet to be tested for HIV prevention in a community-based setting with this population.

**Trial registration:**

ClinicalTrials.gov NCT02437123 https://clinicaltrials.gov/show/NCT02437123 (registered 4 May 2015). Protocol version: 24 July 2015.

## Background

Indigenous scholars suggest that understanding substance use and HIV vulnerability among young Indigenous people must begin with consideration of the ongoing impact of colonization and intergenerational trauma while also acknowledging strength and survival in the face of incredible hardships [1–5]. Indigenous scholars have described intergenerational trauma as a collective emotional and psychological “soul wound” that continues to affect the youngest generations of Indigenous people [6, 7]. In Canada, well over 150,000 Indigenous children were forcibly removed from their homes and placed in residential schools between 1883 and 1996 [8]. The persistent devaluation of Indigenous cultural identity and widespread physical, sexual, and emotional abuse that occurred at residential schools has had far-reaching consequences for the health of Indigenous people [9]. Many Indigenous scholars and advocates argue that cultural oppression through the removal of children from their families and communities has continued through the child welfare system [10]. In fact, estimates indicate that three times as many Indigenous children are currently in care of the state compared with during the peak of the residential school system in the 1940s [10]. In a study of young Indigenous people who use drugs in British Columbia (BC), 65 % had been removed from their biological parents and placed into care [11]. Those who had been in care were 2.1 times more likely to have a parent who attended residential school and 2.6 times more likely to have been sexually abused. The residential school and child welfare systems in Canada disrupted ways of life that had sustained Indigenous families and communities over generations, and introduced cycles of multi-generational grief, trauma, and displacement [10]. Acknowledging the profound effect of intergenerational and lifetime trauma, and subsequent reliance on the powerful numbing effect of drugs, is critical to understanding HIV vulnerability among young Indigenous people who use drugs [4, 7, 12–15].

Indigenous leaders in Canada are justifiably concerned about alarming rates of HIV infection among their young people who continue to be vastly over-represented in the HIV epidemic [16]. Although Indigenous people comprise just 3.8 % of Canada’s total population, Indigenous people in 2013 represented 16 % of positive HIV tests in the country [17]. Injection drug use remains the primary exposure among Indigenous people, accounting for 64 % of HIV infections, compared with 11 % among non-Indigenous people [16]. In addition, one third of new infections were among young Indigenous people under 30 years of age, compared with only 20 % among young non-Indigenous people [16]. There is a continuing crisis of HIV infection among young Indigenous people who use injection and non-injection drugs in BC; HIV prevalence reached 9 % overall and 17 % among those who inject drugs [12]. In addition, HIV incidence among young Indigenous people who use injection drugs in BC is estimated to be three times higher than among non-indigenous young people [18].

Significant and multi-layered barriers to HIV prevention services, including health care, harm reduction, addiction treatment, and safer sex work environments, have been identified among young Indigenous people who use drugs [2, 3, 12, 13]. Young Indigenous people who use drugs have described frequent encounters with systemic and interpersonal racism, stigma and judgement within harm reduction and health services [1, 19–23]. Furthermore, despite compelling evidence that harm reduction programs, including supervised drug consumption facilities [24] and opioid substitution therapy [25], are effective at preventing HIV infection, studies indicate that young Indigenous people are failing to engage with these services [12, 18, 26, 27]. Finally, safety in sex work, as well as supports to leave sex work if desired, remains elusive in a country where young Indigenous women in sex work routinely face extreme violence and death in the course of their work [28, 29].

Addressing barriers to HIV prevention among young Indigenous people who use drugs—while also acknowledging resilience despite adversity—is urgently required. In New Zealand, Maori nurses have emphasized the need for health care that is “culturally safe” in order to overcome power imbalances that shape interactions between health-care providers and Indigenous clients [30]. Culturally safe care simultaneously privileges Indigenous worldviews and acknowledges the impact of structural violence experienced by colonized peoples in health-care settings [31]. Indigenous experts also recognize the importance of “culture as intervention” to support resilience and resistance to HIV infection as well as trauma-informed counseling as a response to the harmful effects of historical and lifetime trauma [1]. Others have noted the critical role of interconnectedness with family and community to support health and healing among Indigenous individuals [32]. Further research has highlighted the importance of non-medical, supplementary support for young Indigenous people in order to break through layers of marginalization experienced in their everyday lives [33].

Mobile health (mHealth), the provision of health care via mobile phones, has been identified as potential tool to support young drug-using Indigenous people to overcome barriers to HIV prevention. Recent trials in sub-Saharan Africa have provided evidence that supportive text-message interventions can improve HIV treatment adherence and viral suppression among people living with HIV [34, 35]. Others have begun to investigate the impact of mHealth for HIV care and treatment among people living with HIV who use drugs [36–38]. However, much of the existing evidence on mHealth interventions for HIV prevention has focused on improving HIV knowledge by delivering HIV prevention messages to diverse at-risk groups [39]. To our knowledge, no studies have investigated the potential for a two-way supportive text-message program to reach out to young drug-using Indigenous people to reduce vulnerability to HIV infection.

This protocol outlines a multi-site Zelen pre-randomized trial to measure the effect of a culturally safe, two-way supportive text-message intervention to reduce HIV vulnerability among young Indigenous people who use illicit drugs. This work will build on findings from recent studies implementing mHealth interventions in HIV clinics in Nairobi, Kenya and Vancouver, Canada [34, 40]. Outcomes include HIV propensity score, HIV risk, resilience, psychological distress, access to drug-related services, and connection to culture. We hypothesize that the Cedar Project WelTel mHealth intervention will result in improved uptake and access to HIV prevention services, including health-care, harm reduction and addictions services, cultural supports, housing, and counseling. We believe that, together with the supportive element that “someone cares” enough to check in each week, improved access to services will help strengthen participants’ resilience and reduce vulnerability to HIV infection.

## Methods and design

### Study design

The Cedar Project WelTel mHealth study is a two-site, two-arm, parallel group, open, stratified Zelen pre-randomized controlled trial to measure the effect of a culturally safe, two-way supportive text-message intervention to reduce HIV vulnerability among young Indigenous people who use illicit drugs in a community-based setting (ClinicalTrials.gov identifier NCT02437123). Participants will be pre-randomized to two groups—either the mHealth intervention or standard care—at a 1:1 allocation ratio.

### Study setting

This trial is a sub-study nested within the Cedar Project, a cohort study of young Indigenous people who use injection and non-injection drugs and reside in Vancouver and Prince George, BC, Canada. Initial recruitment into the Cedar Project occurred between 2003 and 2007 and was re-opened in 2011. To date, 738 participants have enrolled in the main Cedar Project cohort in Vancouver and Prince George. Eligibility criteria included being between the ages of 14 to 30 at enrollment, provision of informed consent, self identification as having Indigenous ancestry, and smoking or injecting drugs—including crystal methamphetamine, opiates, crack or cocaine—at enrollment. Cedar Project participants complete an enrollment visit as well as follow-up visits every 6 months. At each study visit, participants complete detailed questionnaires eliciting demographic, behavioural and health information, administered by trained Indigenous interviewers. Participants also provide a venous blood sample for HIV and hepatitis C virus antibody testing. In gratitude for participants’ time, honoraria are provided at each follow-up visit. The Cedar Project has been approved by the University of British Columbia Providence Health Care Research Ethics Board (H02-50304).

The Cedar Project study storefront research offices are located in Vancouver’s downtown eastside and in the downtown core of Prince George. Vancouver is a large city in southern BC and is located on the traditional territory of the Coast Salish peoples. Prince George is a forestry and mining town in the province’s northern interior, located on the traditional territory of the Lheidli T’enneh people. Cedar Project participants represent many of the diverse First Nations, Inuit and Métis communities across Canada and are often living far away from their home communities. Participants are highly transient, moving frequently between cities and reserves, making continuity of care a tremendous challenge [41]. Indigenous interviewers and nurses support both sites and are encouraged to provide referrals to cultural supports, health care, substance use services, food programs, housing, and counseling for participants who seek them.

### The Cedar Project Partnership model

Since its inception, the Cedar Project has been community-driven, interdisciplinary research that responds to the continuing crisis of HIV infection and contributes to the health and healing of young Indigenous people who use drugs. The Cedar Project Partnership, an independent body of Indigenous Elders, health and social service experts, researchers, and elected leaders, governs the entire research process. The Cedar Project Partnership recognizes that conducting culturally safe research with young, vulnerable Indigenous people requires creating safe spaces where their identities, voices and stories are heard and respected [30]. A critical component of the Cedar Project is to ensure that our site offices are safe, welcoming settings without judgement of drug use, where police are not allowed, and cultural identity is honoured. Part of building cultural safety into the Cedar Project includes supporting access to traditional foods and ceremonies, such as through annual feasts, memorials, and a recent Learning Potlatch to honour our partners and participants held in Prince George. Overall, our paradigm is to acknowledge grief and historical trauma while building on young Indigenous peoples’ strengths.

### Participants

**Fig. 1:**
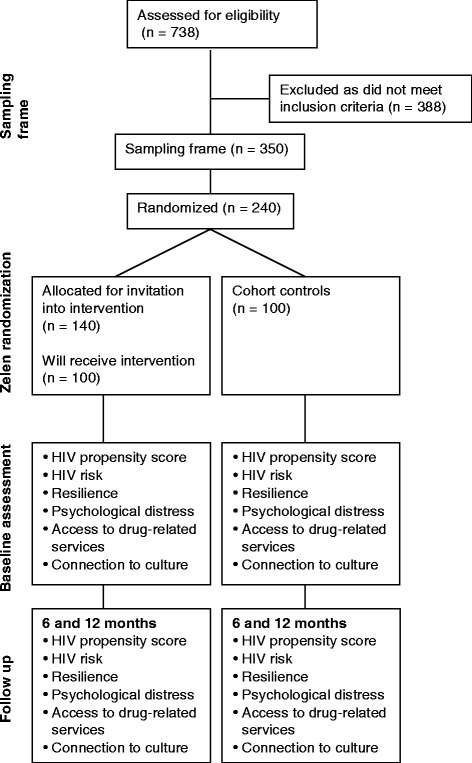
Cedar Project mHealth Study for HIV prevention CONSORT diagram of study design

A stratified random sample of participants in the Cedar Project cohort will be selected for participation in the Cedar Project WelTel mHealth study (Fig. 1). A sampling frame of eligible Cedar Project participants will be created on the basis of the following eligibility criteria:

1.  Currently enrolled in the Cedar Project
2.  Completed main Cedar Project baseline questionnaire and attended at least one follow-up visit since 2009
3.  Had not tested positive for HIV
4.  Joined study in Vancouver or Prince George
5.  Alive at initiation of the Cedar Project WelTel mHealth study.

Participants who do not meet the eligibility criteria or who decline to participate will be excluded. Stratified randomization in a 1:1 ratio will be used to ensure a balance of key factors, including study site (Prince George and Vancouver) and reporting injection drug use at the last visit (yes versus no). Sampled participants will be randomly assigned to receive the intervention or be included in the comparison group through computer-generated codes developed by a statistician at the Centre for Evaluation and Outcome Sciences at St. Paul’s Hospital. A list of participants to be invited into the intervention arm will be distributed to each study site. Informed consent will be sought from each participant invited to receive the intervention who agreed to participate. Those assigned to the comparison group will continue in the usual Cedar Project cohort study under its existing informed consent with no change whatsoever to their participation in the overall study. Target enrollment is set at 200 participants.

#### Intervention group

To ensure that we reach our target sample, initial randomization will be slightly unbalanced to accommodate refusals to participate or difficulty locating participants. A total of 140 participants (70 from each site) will be randomly allocated to receive the invitation to participate in the intervention arm of the mHealth study. This will allow for an estimated 20 participants per site who cannot be reached or who decline to participate. Cedar staff will invite and enroll participants from this list to join the study until enrollment reaches 50 at each study site. Three attempts will be made to invite participants in the intervention arm into the study, including during regular visits to the research offices, phone, email, word of mouth, and street outreach.

#### Comparison group

One hundred Cedar Project participants who meet the eligibility criteria will be allocated to serve as the cohort comparison group, stratified on the same variables used to select the intervention group. Participants randomly assigned to the control group will continue as Cedar Project participants under usual circumstances. This group of cohort controls will not be required to provide any additional data to the study beyond that which they provide in semi-annual follow-up interviews as participants in the main Cedar Project study.

### The intervention

The Cedar Project WelTel mHealth intervention being evaluated is a structured mobile-phone initiative to connect young Indigenous people who use drugs with Cedar Case Managers in a community-based setting. The intervention consists of a package of supports, including a mobile phone and cellular plan, weekly two-way text messaging, and support from Cedar Case Managers. The intervention will be implemented over a 16-month period, and all participants will receive the intervention for a minimum of 6 months.

Cedar Case Managers include Indigenous and non-Indigenous nurses and Cedar Project staff members who have extensive frontline outreach experience with young Indigenous people who use drugs in Prince George and Vancouver. Cedar Case Managers follow a “culturally safe” approach that acknowledges both trauma and strengths and includes an explicit focus on the critical roles of cultural assets and safe relationships with care providers in HIV risk reduction [31].

Participants allocated to receive the intervention will be provided with a cellular phone at the start of the study. Each phone is pre-programmed with several phone numbers for emergency and health-related services relevant to the study site. During the study period, participants will receive a monthly cellular plan that includes unlimited calling and texting within Canada, but no data. We made an explicit decision to include long-distance calling and texting to provide the opportunity for participants to connect with family and loved ones living in home communities and elsewhere in Canada. If phones are lost or stolen, participants will be eligible to receive one replacement. If two phones are lost, the participant may bring their own sim card-enabled cellular handset to use with the study monthly cellular plan.

**Fig. 2:**
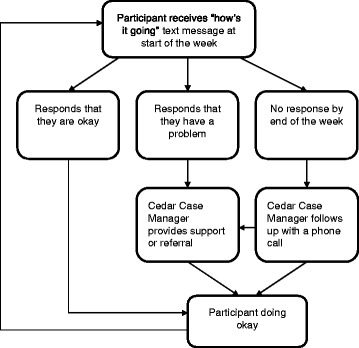
The Cedar Project WelTel mHealth intervention

The supportive text-message component of the study is based on the intervention tested in the WelTel Kenya1 trial (Fig. 2) [34]. Each Monday at noon, a text message saying, “How’s it going?” is automatically sent to intervention arm participants through the WelTel mHealth software platform. Cedar Case Managers will login within 24 to 48 hours to triage the incoming text messages. They will respond to all participants and follow up with participants who replied with a specific problem or need. On Wednesday, participants who have not replied will receive an additional text saying, “Haven’t heard from you, are you OK?” On Thursday or Friday, staff will attempt to call all remaining participants who have not responded by text message. Throughout, study staff will keep a log of all responses and actions taken through the WelTel platform.

### Blinding

We recognize that blinding in a trial such as ours is challenging. Care providers (Cedar Case Managers) and intervention arm participants cannot be blinded, as the mHealth intervention requires overt participation and an “invite list” is provided upfront. However, participants in both the intervention and control groups will be blinded to specific study hypotheses. Primary and secondary outcomes will be drawn from routine data collection that occurs as part of participation in the overall cohort; it is unlikely that participants will identify the specific questions used to generate outcomes from our extensive questionnaires. The data analyst will also be blinded to group allocation.

### Objectives

The study’s primary objective is to test whether the Cedar Project mHealth intervention reduces vulnerability to HIV among young Indigenous people who use drugs, measured by using an HIV propensity score described below. Our secondary objectives are to test the specific ways the mHealth intervention may reduce vulnerability to HIV by exploring HIV risk behaviours, resilience, psychological distress, access to drug-related services, and connection to culture among participants over the course of the study.

We hypothesize that participants receiving the intervention will seek help from Cedar Case Managers to navigate barriers to drug-related services, housing, cultural supports, and health care. Also, we hypothesize that participants will use the phones as tools to connect with other supports and sources of strength in their lives, including family, work, culture, and services. Thus, we hypothesize that the intervention will help young people to work towards goals related to their own health and well-being, supporting them to reduce HIV risk.

### Outcome measures

#### Primary outcomes

The primary outcome (Table 1) is HIV risk, measured by an HIV propensity score assessed at the 6-month time point. Previous analyses of Cedar Project data with respect to HIV infection have identified several factors as associated with HIV infection, including recent injection drug use, high-frequency drug use, needle sharing, and participation in sex work [12]. Logistic regression of risk factors for prevalent HIV infection at study recruitment will provide coefficients to combine these four risk variables into a single primary outcome measuring propensity for HIV infection. Mean change in HIV propensity score from baseline to 6 months will be used to determine the impact of the intervention on HIV vulnerability.

**Table 1:** Outcome measures

Outcome measure	Specific variable	Analysis metric	Hypothesis (intervention > control)	Type	Analysis
Primary					
HIV propensity (6 months)	HIV propensity score	Mean change - HIV propensity score	Decrease in HIV risk	Continuous	T test
Secondary					
HIV propensity (12 months)	HIV propensity score	Mean change - HIV propensity score	Decrease in HIV risk	Continuous	T test
HIV vulnerability	Injection drug use	Mean change - proportion injection	Decrease in injection	Binary	χ^2^ test
	High-frequency injection	Mean change - proportion frequent injection	Decrease in injection frequency	Binary	χ^2^ test
	Needle sharing	Mean change - proportion needle sharing	Decrease in needle sharing	Binary	χ^2^ test
	Participation in sex work	Mean change - proportion sex work	Decrease in sex work	Binary	χ^2^ test
Resilience	Connor-Davidson Resilience Scale (CD-RISC)	Mean change - CD-RISC score	Increase in resilience	Continuous	T test
Psychological distress	Symptom Checklist-90-R (SCL-90-R)	Mean change - SCL-90-R score	Decrease in psychological distress	Continuous	T test
Access to drug-related services	Opioid substitution therapy (OST)	Mean change - proportion accessed OST	Increase in access OST	Binary	χ^2^ test
	Needle exchange (NX)	Mean change - proportion attended NX	Increase in access NX	Binary	χ^2^ test
	Safe injection facility (SIF)	Mean change - proportion visited SIF	Increase in access SIF	Binary	χ^2^ test
	Drug treatment	Mean change - proportion seeking treatment	Increase in seeking treatment	Binary	χ^2^ test
	Tried to quit using drugs	Mean change - proportion trying to quit	Increase in trying to quit	Binary	χ^2^ test
Connection to culture	Participation in traditional ceremonies	Mean change - proportion participated in traditional ceremony	Increase in access to ceremony	Binary	χ^2^ test
	Frequently living by traditional culture	Mean change - proportion lived by traditional culture	Increase in traditional culture	Binary	χ^2^ test

All variables refer to behaviour in the 6-month period preceding the interview. All variables are measured as mean change in proportion or score from baseline

#### Secondary outcomes

The potential benefits of the supportive mHealth intervention are broad. As a result, multiple secondary outcomes are of interest. These were selected through consultation with academic and community partners and are part of routine Cedar Project data collection. They fall under five categories: HIV risk, resilience, psychological distress, access to drug-related services, and connection to culture. For all secondary outcomes, the effect of the mHealth intervention will be measured as change in the outcome from baseline to 6 months. Participants with sufficient follow-up time will contribute to analyses of all outcomes at the 1-year time point to determine longer-term effects of the intervention.

##### HIV propensity (12 months)

Participants who received the intervention for a sufficient length of time will contribute to a secondary analysis of change in HIV propensity at 1 year to determine longer-term effects of the Cedar Project WelTel mHealth intervention. The HIV propensity variable will be generated by using the same approach used for the primary outcome.

##### HIV vulnerability

Although the effect of the intervention on HIV risk will be assessed in the primary analysis, we will also analyze the effect of the intervention on specific behaviours. Several self-reported binary measures will be used to determine the effect of the intervention on HIV vulnerability in the previous 6-month period, including injection drug use in the past 6 months, high-frequency (daily or more) drug use, needle sharing, and participation in sex work. These measures will be ascertained from Cedar Project questionnaires collected every 6 months as part of the main Cedar Project cohort. HIV vulnerability will be assessed at baseline, 6 months, and 1 year.

##### Resilience

Resilience, or the ability to cope with adversity, will be characterized by using the Connor-Davidson Resilience Scale (CD-RISC). The creators of the scale broadly define resilience as “personal qualities that enable one to thrive in the face of adversity” [42]. The CD-RISC scale measures resilience via 25 items on a five-point scale with scores ranging between 0 and 100, with higher scores indicating greater resilience. Resilience will be assessed at baseline, 6 months, and 1 year.

##### Psychological distress

The Symptom Checklist-90-R (SCL-90-R) is a 90-item self-reported symptom inventory that measures the severity of nine dimensions of psychological distress in the past 3 months scored on a five-point Likert scale (from ‘not at all’ to ‘extremely’). Participants’ SCL-90-R scores will be transformed into an average Global Severity Index, providing a single average measure that profiles overall degree of psychological distress [43]. Psychological distress will be assessed at baseline, 6 months, and 1 year.

##### Access to drug-related services

Self-reported access to drug-related services, including opioid substitution therapy, needle exchange, safe injection facility, and drug treatment, in the previous 6-month period, will be ascertained from the main Cedar Project questionnaire. Proportions of participants reporting access to these services will be compared in the intervention and control groups. We will also determine whether there are differences among treated and control groups in terms of proportion of people who tried to quit in the previous 6-month period. Access to drug-related services will be assessed at baseline, 6 months, and 1 year.

##### Connection to culture

Connection to Indigenous culture has been hypothesized as a key protective factor for young Indigenous people who use drugs. It will be assessed by using two dichotomous variables that measure cultural activity in the prior 6-month period: (1) self-reported participation in traditional ceremonies (including potlatch, feast, fast, burning ceremony, washing ceremony, naming ceremony, big/smoke house, rites of passage, smudge, dances, or any other traditional Indigenous ceremony) and (2) frequency living by traditional culture (never/rarely versus often/always). These variables were defined by Earl Henderson (Cree, Métis) and Violet Bozoki (Lheidli T’enneh) who are Indigenous Elders, traditional knowledge keepers, and members of the Cedar Project Partnership.

### Sample size

Considering current follow-up rates, we are likely able to reach and recruit at least 200 participants for participation in both arms of the Cedar Project WelTel mHealth study. As mentioned previously, the primary objective is HIV risk, measured by using an HIV propensity score evaluated at 6 months. On the basis of a 1:1 allocation ratio, a significance level of 0.05 (one-sided) and 80 % power, it is estimated that we require 78 participants in each study arm. This computation assumes a standardized mean effect size (using Cohen’s d) of 0.40. Assuming attrition rates of approximately 20 %, we intend to over-enroll, resulting in a target of 200 participants overall (100 in each arm). Sample size was calculated by using the pwr package in R, software version 3.2.1.

### Statistical analysis

The analysis and reporting of the results will follow the CONSORT (Consolidated Standards of Reporting Trials) guidelines [44]. Descriptive statistics of socio-demographic, historical/lifetime trauma, sex- and drug-related HIV vulnerabilities, and health-related variables will be presented to assess comparability of the intervention and control groups. Mean (standard deviation) or median (quartiles) will be used for continuous variables and count (percentage) for categorical variables. Our primary analysis to evaluate the effect of the mHealth intervention on HIV prevention will be by intention to treat (ITT) for primary and secondary outcomes among all randomly assigned participants according to the study group to which they were originally allocated. Student’s *t* tests and chi-squared tests will be used to determine differences in the mean change in proportions or scores from baseline at 6 months or 1 year between the two study arms. If baseline characteristics are found to be substantially different between the two study arms, we will adjust for these factors by using regression models. In addition, we will conduct a secondary analysis by using a modified ITT approach including only participants who agreed to participate in the intervention making up the intervention group (excluding over-sampled participants who did not receive the intervention). To minimize the risk of bias introduced in our modified ITT analysis, we propose to use complier average causal effect (CACE) analysis [45]. This approach will allow us to retain the initial random assignment by taking into account two subgroups within the group pre-randomized to receive the intervention (those who did and did not actually receive the intervention) and then helping us to identify a similar subgroup of control participants who could have been expected to receive the intervention had it been offered [45]. By comparing expected and observed outcomes in these subgroups, we will obtain a less biased estimate of effect of the intervention [45]. Missing data will be approached by using multiple imputation. An up-to-date version of R statistical software will be used to conduct all analyses [46]. All tests will be two-sided; *P* values of less than 0.10 will be considered significant. We plan to conduct four predetermined exploratory subgroup analyses that may help to tailor the intervention for specific high-need populations, including comparisons by gender, city, injection drug use, and urban versus rural. Statistical methods similar to those described above for whole-group analyses will be conducted for primary and secondary outcomes. All subgroup results will be reported, regardless of significance. Inferences will be hypothesis-generating, given concerns related to multiple comparisons and lower power to detect an effect in subgroups.

### Nested studies

#### Study implementation

As this study is the first of its kind in a community-based setting among young Indigenous people who use drugs, it is critical that the implementation of the intervention be described in detail. Daily mHealth memos prepared by each study site, combined with procedural documents, will inform a description of how a supportive two-way text-message program looks and functions in practice. Descriptive statistics generated from the mHealth baseline questionnaire will provide additional insight into the feasibility of applying this program in similar settings. The survey asks about ownership and use of mobile phones, how often participants text message, and perceived helpfulness or concerns about receiving text messages related to health.

#### Types of support requested

Examining the types of support requested by Cedar Project WelTel mHealth participants via text will help to identify current challenges accessing prevention services and support among young Indigenous people who use drugs. A comparative content analysis of text-message interactions captured on the WelTel platform over the study period will be used for this purpose. This descriptive analysis will help inform scale-up of this program, including the human resource requirements for future implementation in similar settings.

#### Participant perceptions

Participant perceptions of the mHealth intervention will be evaluated via the mHealth follow-up questionnaire. All intervention arm participants will be invited to complete an administered questionnaire to assess satisfaction with the care and support they received via the intervention. Evaluation of patient perceptions will include thematic analysis of narrative responses and basic descriptive statistics.

### Ethics

The Cedar Project follows the guidelines provided in the Tri-Council Policy Statement on Ethical Conduct for Research Involving Humans – Chapter Nine: Research involving the First Nations Inuit and Métis Peoples of Canada [47]. In addition, the study will adhere to the principles of Ownership, Control, Access and Possession in relation to research with Indigenous people [48]. Through the Cedar Project Partnership, Indigenous collaborators will continue to be involved in the conception, design and interpretation of the results of the Cedar Project WelTel mHealth study. The Cedar Project Partnership will serve as the data and safety monitoring board for this study. They have also approved this manuscript for publication. The Cedar Project WelTel mHealth study has been approved by the University of British Columbia Providence Health Care Research Ethics Board (H13-02718), and we will be accountable to them for approval and monitoring.

All participants have provided informed consent as part of the main Cedar Project cohort study. Participants randomly assigned to receive an invitation to participate in the Cedar Project WelTel mHealth intervention will undergo an additional consent process. After the study participant is invited to participate in the Cedar Project WelTel mHealth study, a trained Cedar Project staff member will describe the study. If the person would like to enroll, the research staff will review the consent form and answer any questions. Participants who give consent are provided with a copy of the study introduction letter and consent form.

### Harms

Adverse events will be documented in writing and reported to investigators. Study staff at both sites have been trained in recognizing and reporting of adverse events, including those directly attributable to the intervention (e.g., accidental disclosure of illicit drug use) and those resulting from trial participation. Because the study takes place in a community-based (not clinical) setting, staff will make referrals to care where appropriate. Potential harms will be outlined to participants during the informed consent process. All unanticipated risks to human participants or others will be reported to the Cedar Project Partnership and the University of British Columbia/Providence Health Care Research Ethics Board where appropriate.

### Dissemination

Knowledge translation is a hallmark of the Cedar Project and will be a key component of the Cedar Project WelTel mHealth study. Integrated knowledge translation is planned throughout the study, and updates will be provided to the Cedar Project Partnership at quarterly meetings. Findings will be shared through ceremony with Indigenous partners; peer-reviewed publications and presentations; and consultations with policy makers and service delivery organizations to support uptake of results.

## Discussion

Innovative, culturally safe interventions that address the barriers to HIV prevention while supporting the strength of young Indigenous people who use drugs are urgently needed. This study, due to report its findings in 2017, tests the effectiveness of a two-way supportive text-message program delivered in a community-based setting to support HIV prevention among young Indigenous people who use drugs. Despite presenting a tremendous opportunity to connect young, highly transient Indigenous people who use drugs to prevention services and support, supportive two-way mHealth programs have yet to be tested for HIV prevention in a community-based setting with young Indigenous people who use drugs [34, 49–51].

Other researchers have used two-way supportive messaging interventions to engage people living with HIV in care and to improve adherence [34, 36, 40, 52, 53]. Recent randomized controlled trials in Kenya indicated that introducing mobile-phone technology into HIV case management improved HIV treatment adherence and HIV-related clinical outcomes, despite high levels of poverty, remote and rural living, transience, stigma, and discrimination [34, 50, 54]. These studies have demonstrated the importance of text-message programs that are both supportive and interactive [35, 55–57], as passive mHealth programs that seek to monitor, remind or educate have had limited success [58–60]. It is important to note that we have deliberately chosen an open-ended weekly text message to allow participants to set their own priorities around their health and well-being, as well as to avoid possible disclosure of drug use and other sensitive issues.

We have chosen a cohort-embedded Zelen pre-randomized controlled trial design [61–63]. Our ongoing Cedar Project cohort study provides the opportunity to support recruitment into the embedded trial and also allows us to use routinely measured outcomes collected longitudinally among the whole cohort. All participants entering the cohort have consented to providing observational data. Consent to “try” the intervention is being sought only from those who have been pre-randomized to receive the intervention [63]. Study outcomes among randomly selected participants will be compared with those of the cohort members not randomly selected to receive the intervention. Another advantage of this design is to minimize false hopes, resentment, and contamination among controls had they learned they were not receiving a potentially valued intervention through trial consent and randomization [61, 62]. In our instance, as it is not possible to completely separate intervention participants from the rest of the cohort, it is likely that some members of the cohort will come to know about the intervention. However, even if control participants have or obtain a phone and cellular plan, full crossover of participants from control to intervention is not possible, owing to the nature of the intervention. As noted above, neither group will be aware of the hypotheses under study. A limitation of this design is that a significant number of participants allocated to the intervention arm may refuse to receive it whereas control subjects do not have this option. Including the former group in an ITT analysis may dilute the treatment effect, whereas excluding them could lead to a biased comparison. In our case, we believe that the intervention is likely to be highly valued among invitees and there will be few who refuse. Nevertheless, this will be monitored closely, and as noted earlier, we will also use a modified ITT approach.

A key component of the Cedar Project WelTel mHealth study is relational accountability [64]. We honour our relationships with study participants who have continued to share their stories with us since joining the Cedar Project. Testing this intervention is a way for us to respond to the clear message from Cedar Project participants about the importance of having support to navigate barriers to care and services that they face in their daily lives. An additional component of our relational accountability is through the governing leadership of the Cedar Project Partnership, who work to ensure that this research is relevant to the communities they represent and is conducted in a good way.

### Limitations

Our decision to over-sample in the intervention arm to adjust for refusals to participate or difficulty locating participants may result in a dilution of the effect of the intervention in ITT analysis. As a result, we have proposed a parallel, modified ITT analysis, which may be affected by selection bias. Diversity of HIV risk among participants (non-homogeneity) may also dilute the detected effect of the intervention. Representativeness of the sample is dependent on representativeness of the Cedar Project cohort study sample overall; however, we have made a considerable attempt to ensure that the sample represents the population under study [12]. As this study involves a package of support that includes mobile phones, two-way text messaging, and support, it is difficult to determine the relative contribution of components of the package to any effect the intervention may have. The qualitative nested studies will help in this regard. Furthermore, we cannot rule out that simultaneous co-interventions could impact our results. As BC’s HIV incidence has been stable for several years and many public health initiatives (including needle exchanges and safe injection sites) are already in place, we feel it is unlikely that new transformative co-interventions will take effect over the study period. However, we will monitor potential co-interventions that could impact study outcomes through our main cohort questionnaires, as well as track major policy and programmatic changes in the Province. Blinding in this study is not possible as assessors have strong relationships with participants. In addition, loss to follow-up may be higher in the comparison group than the intervention group. Cedar Project participants are highly transient and often difficult to track down [41]. Having a mobile phone and consistent airtime will likely make it easier to reach participants receiving the intervention for follow-up visits. Active follow-up with all participants through phone, email, and outreach will help ensure retention and interview appointment attendance. In addition, our measures may not adequately capture complex concepts such as connection to culture and resilience. Finally, the intervention under study is complex and multifaceted. It may be that our proposed outcomes do not adequately capture the impact of the intervention on the lives of participants involved.

### Trial status

Enrollment in the trial has begun but has not yet reached full enrollment.

## Abbreviations

BC: British Columbia
CD-RISC: Connor-Davidson resilience scale
ITT: Intention to treat
mHealth: Health care delivered via mobile phone
NX: Needle exchange
OST: Opioid substitution therapy
SIF: Safe injection facility
SCL-90-R: Symptom checklist 90-R
